# Ultraviolet germicidal irradiation for surface cleaning of COVID-19 in healthcare settings: A review

**DOI:** 10.4102/jphia.v16i2.572

**Published:** 2025-02-28

**Authors:** Chioma M. Oringanje, Sidney K. Oparah, Chukwudi Oringanje, Chibuike M. Meremikwu, David Olatunji, Alice A. Uzuta, Chinwe L. Ochu

**Affiliations:** 1 Department of Biology, College of Arts and Science, Xavier University, Cincinnati, United States of America; 2 Department of Internal Medicine, University of Calabar, Calabar, Nigeria; 3 Cochrane Nigeria, Institute of Tropical Diseases, Research and Prevention, University of Calabar Teaching Hospital, Calabar, Nigeria; 4 Institute of Tropical Diseases Research and Prevention, University of Calabar Teaching Hospital, Calabar, Nigeria; 5 Nigeria Centre for Disease Control and Prevention, Abuja, Nigeria; 6 George Washington University, Washington, DC, United States of America

**Keywords:** ultraviolet germicidal irradiation, COVID-19, Coronavirus, disinfection, healthcare

## Abstract

**Background:**

The COVID-19 pandemic led to the implementation of additional infection prevention and control (IPC) measures. In healthcare settings, the risk of severe acute respiratory syndrome coronavirus-2 (SARS-CoV-2) infections remains high for patients, healthcare workers, and visitors. Ultraviolet germicidal irradiation (UVGI) has been explored as a potential alternative for surface disinfection within healthcare facilities and hospitals.

**Aim:**

This study evaluates the effectiveness of UVGI as a surface cleaning method for COVID-19.

**Setting:**

Healthcare settings.

**Method:**

A systematic literature review was conducted following PRISMA guidelines. Databases searched from 01 January 2020 to 31 August 2022, included Cochrane Central Register of Controlled Trials (CENTRAL), MEDLINE, Embase, and Cochrane Database of Systematic Reviews (CDSR), with no language restrictions. Two independent researchers screened and extracted data. Proportions and relative risk were calculated, and the evidence quality was assessed using the GRADE approach.

**Results:**

Three studies were included, all focusing on terminal disinfection of patient rooms. None directly assessed the effect of UVGI on hospital-acquired SARS-CoV-2 infections. One study found UVGI reduced viral contamination post-regular cleaning in healthcare settings (RR: 1.83, 95% CI: 1.02–3.31). Other studies reported complete viral ribonucleic acid (RNA) clearance after 15 min of irradiation at 254 nm and 15 s at 222 nm, respectively.

**Conclusion:**

The evidence on UVGI reducing SARS-CoV-2 contamination on surfaces is of very low certainty.

**Contribution:**

The very low certainty prevents a definitive conclusion on its effectiveness in preventing COVID-19 in healthcare settings. Further research is needed to strengthen the evidence base.

## Introduction

Coronaviruses (CoVs) are a large family of viruses that can cause illness, ranging from the common cold to more severe diseases such a Middle East Respiratory Syndrome (MERS-CoV) and the severe acute respiratory syndrome coronavirus (SARS-CoV).^1^ The novel coronavirus (SARS-CoV-2) is a new strain from the CoV family.^2^ Because of the contagious nature of the disease, limiting the transmission of SARS-CoV-2 is a critical component of patient care for those with suspected or confirmed COVID-19. To prevent the spread of SARS-CoV-2, a combination of strategies is employed, including source control measures such as wearing masks to contain respiratory secretions, early identification and isolation of infected persons, the use of appropriate personal protective equipment (PPE), frequent hand washing, chemical disinfection, filtration, heat sterilisation and proper ventilation.^3,4^

Ultraviolet germicidal irradiation (UVGI) is an established means of disinfection and can be used to prevent the spread of infectious agents. First used in a healthcare setting in 1936 at Duke Hospital, North Carolina,^5^ UVGI is an effective means of inactivating microbes using ultraviolet (UV) energy. Ultraviolet germicidal irradiation specifically utilises UV-C light, which falls within the germicidal wavelength range of 200 nm – 280 nm, with the 250 nm – 270 nm range being the most effective germicidal wavelength of light.^6^ Compared to UV-A and UV-B, UV-C poses less risk to human health, as it is less penetrating.^7,8^ Ultraviolet germicidal irradiation can be used for both air and surface disinfection.^7^ Upper room UVGI, one of the primary applications of UVGI air disinfection has been in use for over 70 years in controlling the spread of tuberculosis.^9^

Ultraviolet C (UVC) light emitted by UVGI systems works by preventing the transcription and replication of microorganisms, leading to their inactivation.^6^ A primary application of UVGI for air disinfection is in-duct UVGI, where air is disinfected as it passes through the heating, ventilating and air conditioning (HVAC) system before being recirculated. This method irradiates the entire cross-section of the duct at high intensities which are not accessible to room occupants. In addition to air disinfection, UVGI is used to disinfect surfaces within HVAC systems, such as cooling coils and drip pans, which may help reduce nonspecific building-related illnesses.^10^ Most conventional prevention and control methods rely on contact disinfection using disinfectants such as 70% ethyl alcohol and 0.1% bleach. Frequent use of some of these methods, heat sterilisation and chemical disinfectants, can damage surfaces, coupled with the severe shortage that was experienced at the peak of the pandemic. Investigators have shown that UVGI can inactivate a range of viruses, including SARS-CoV-2.^7,10,11,12^ Despite its widespread use in other applications like water disinfection, food processing and tuberculosis control, there is limited comprehensive data on the effectiveness of UVGI for inactivating SARS-CoV-2 on surfaces.^13,14,15,16^ This review aims to assess the effectiveness of ultraviolet germicidal irradiation as a surface cleaning measure in healthcare settings to prevent the spread of COVID-19.

## Methods

The present systematic review was conducted in agreement with the Preferred Reporting Items for Systematic Reviews and Meta-Analyses (PRISMA) guidelines for reporting systematic reviews and meta- analyses of randomised controlled trials.^17^

### Literature search

We performed a systematic search of the following electronic databases from 01 January 2020 to August 31, 2022: Cochrane Central Register of Controlled Trials, MEDLINE, Embase and Cochrane Database of Systematic reviews (CDSR). The search strategy consisted of keywords relevant to the intervention, that is UVGI as an environmental (surface) cleaning measure in healthcare settings to prevent COVID-19. We read through the reference lists of relevant studies for articles that may have qualified for inclusion in the review. We applied no language restrictions. The search terms can be found in the Appendix 1 Table 1-A1.

#### Inclusion and exclusion criteria

The review intended to include randomised controlled trials (RCTs) or quasi RCTs that compared UVGI (UV-C energy in the wavelengths: 200 nm – 280 nm) in combination with standard cleaning with other cleaning measures in any healthcare setting under operational conditions. In the absence of RCTs, we considered the following types of study: non-randomised comparative trials and non-control before and after studies. All types of hospital settings, intensive care unit (ICUs), non-ICUs and units provided specialty care were included. All exposure surfaces in the healthcare setting were included; rooms and surfaces, including floors, furniture and equipment in a healthcare setting.

The main outcome was SARS-CoV-2 infection (COVID-19 morbidity rate among healthcare workers and persons in the facilities). Secondary outcomes assessed include clearance or absence of SARS-CoV-2 virus on surfaces in a healthcare facility, optimal irradiation dose and time, and adverse effects associated with the use of UVGI in healthcare setting.

We excluded studies that were carried out in a research laboratory that involve artificial spiking of surfaces and studies that assessed bactericidal efficacy and/or decontamination of personal protection equipment.

#### Study selection

Two team members independently screened titles, abstracts and the full text of relevant articles using the pre-specified inclusion and exclusion criteria. A third team member resolved disagreements.

#### Data extraction and synthesis

Two team members independently collected data on study design, background information on the location and context of the study and any demographic information if available such as, type of health facility, hospital units such as ICU, wards, isolation rooms and so forth, UV source and wavelength, intensity and duration of exposure, mode of assessment. We extracted all relevant data pertaining to the specified outcomes using a data-extraction form. Discrepancies were resolved through discussion with other authors. The risk of bias was assessed for each outcome using a Cochrane Risk of Bias Assessment Tool for Non-Randomised studies of Intervention (ACROBAT-NRSI).^18^

The statistical analyses were performed according to the guidelines set by the Cochrane Collaboration.^19^ Data were analysed using RevMan 5.3 (The Nordic Cochrane Center, Copenhagen, Denmark). We were unable to pool the data from the included studies because of the substantial heterogeneity ranging from different study type, the type of intervention (varying wavelengths of UVGI), the source of UV-C and the exposure times. There were differences in the frequency of sampling as well. We reported crude results without any adjustments. The results were presented in narrative summaries based on the synthesis without meta-analysis (SWiM) guidelines and where possible, the risk difference was presented in a forest plot without meta-analysis.^20^ We evaluated the quality of the evidence for each outcome using the Grading of Recommendations Assessment, Development and Evaluation (GRADE) approach against five criteria: study limitations, inconsistency of effect, indirectness, imprecision and publication bias. The effects and quality of evidence were summarised in GRADE evidence tables.

The protocol of this review was registered with the International Prospective Register of Systematic Reviews (PROSPERO) with registration number CRD42022356495.

### Ethical considerations

This article followed all ethical standards for research without direct contact with human or animal subjects.

## Results

### Study characteristics

**FIGURE 1:**
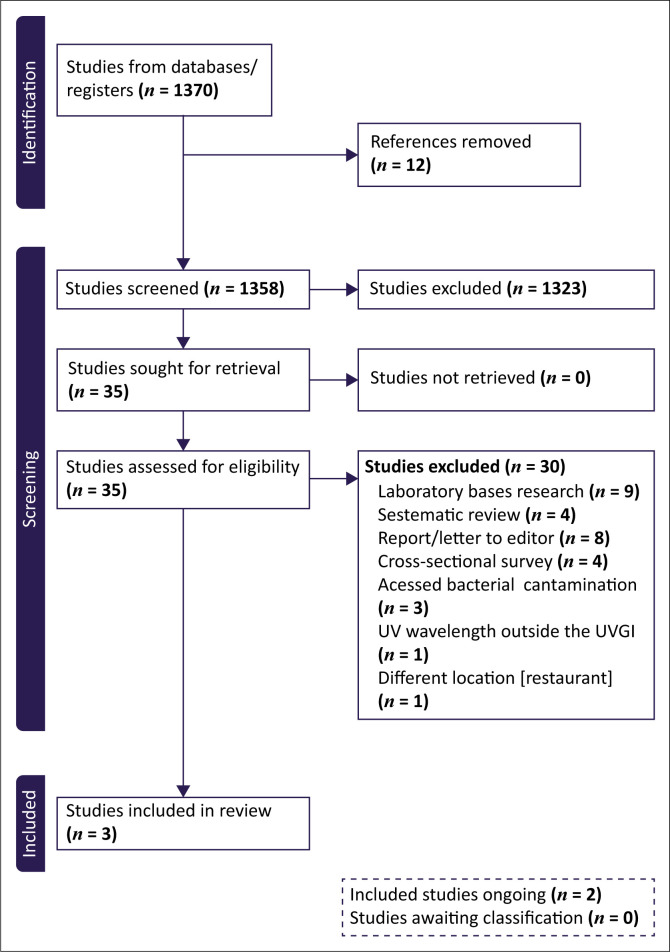
Preferred Reporting Items for Systematic Reviews and Meta-Analyses (PRISMA) flow diagram for study selection.

In total, the search yielded 1370 articles. After de-duplication, 1358 titles and abstracts were screened for eligibility followed by assessment of the full text of 35 potentially eligible studies. Out of these, three studies were included (Table 1).^21,22,23^ Thirty studies were excluded and the reasons for exclusion are described in the Characteristics of excluded studies tables (Online Appendix 1 Table 1). Two ongoing studies were identified (Online Appendix 1 Table 2). The details of the search results are presented in a PRISMA flow diagram (Figure 1).

**TABLE 1:** Characteristics of included studies.

Included studies	Method	Participants	Intervention	Outcome	Comments/notes
Su^22^	Determine the optimal UVC dose required to inactivate SARS-CoV-2 in a clinical hospital setting. Cross-sectional sampling comparing ultraviolet C (UVC) (254 nm) at different irradiation times	Environmental samples were collected from wards where COVID-19 patients with nasal swab of SARS-COV-2 with CtV < 25 were admitted for at least 3 days (ICU and the ward)	UVC (254 nm) irradiation was performed by a hyperlight robot at 5 min, 10 min, 15 min, 20 min and 30 min with estimated energy doses of 42 mJ/cm^2^, 84 mJ/cm^2^, 126 mJ/cm^2^, 168 mJ/cm^2^ and 252 mJ/cm^2^, respectively. Twelve locations in the isolation ward were selected for environmental sampling before cleaning.	Contamination rate (%): number of positive findings divided by total number of environmental samplings multiply by 100 at different irradiation dose	Study date: Not provided **Location**: Taiwan **Sample collection**: Samples were collected from 8–10 sites before routine cleaning using sterile throat swabs and transported in a viral transport medium. Samples were collected from three different locations on each surface of the equipment (a total of 15 cm^2^ area). The sampling was randomly done Samples were collected again after UVC irradiation Sample was analysed using RT-PCR (targeting RNA-dependent polymerase [RdRP] and the E and N genes) Room characteristics: For the rooms, the negative-pressure differences ranged from -14 Pa to – 15 Pa between the ward and buffer areas and – 8 Pa to – 9 Pa between the clean corridor and buffer area. Air exchange was performed 12 times per hour.
Lesho^21^	Evaluated the effectiveness of UV-C irradiation compared to electrostatic spraying or both UV-C and electrostatic spraying in an acute care facility in hospitals (ACH)	Samples from eight stationary surfaces were randomly collected from acute care hospital (ACH) Surfaces included bed rails, call button or remote control, in-room telephone, over-bed tray table, sink and soap dispenser, chair, windowsill, and the floor	Terminal cleaning (wiping) followed by ultraviolet light (UV-C treatment at 60 000 mJ/cm^2^) or electrostatic spray (Clorox1Total 3601), or a combination of both	Reduction in environmental contamination with SARS-CoV-2 RNA	Date: 15 May 2020 – 15 June 2020 **Location:** New York State, US **Sample collection:** Swabs were streaked over targeted high touch surfaces for a minimum of 20 seconds in a rolling motion to ensure contact with the entire swab surface. At the ACH, immediately after a SARS-CoV-2 infected patient was discharged or transferred out of their room, the same eight stationary surface types sampled in the random assessment were sampled immediately before and after terminal cleaning involving surface wiping with a non-bleach sporicidal disinfectant containing hydrogen peroxide and peracetic acid (OxyCide™). Viral RNA was detected via RT-PCR **Room characteristics**: In ACH, shared room configuration involves two beds in the same room separated by a curtain and having a single bathroom for two occupants. In LTCF, two occupants share a single bathroom, but the room is larger and separated by a partial wall, curtain or no physical barrier depending on the unit. Two sampling schemes were applied: random and controlled. In the random subgroup, stationary surface types were sampled at random timepoints in-between cleaning rounds while in the controlled group, the same eight stationary surface types sampled in the random assessment were sampled immediately before and after terminal cleaning that involved surface wiping. The controlled group also involved additional cleaning intervention applied as enhancements over surface wiping. The review focused on data from the controlled subgroups.
Su^23^	A preliminary study to assess ultraviolet C (UVC) irradiation for SARS-CoV-2 on contaminated hospital environments	Swabbed samples from rooms of infected SARS-CoV-2 persons with reverse transcriptase polymerase chain reaction (RT-PCR) cycle threshold values (CtV) lower than 25. All patients (3) in the room presented with bronchopneumonia and stayed in the room without a mask	222-nm UVC irradiation using the Care222 U+ handheld lamp with an energy dose of 27 mJ/cm^2^ with a fix length of 2 cm with stabilised UV radiation intensity. Dosage-related cleaning effect was carried out at 5 s, 10 s, 15 s, and 20 seconds at 27 mJ/cm^2^, 54 mJ/cm^2^, 81 mJ/cm^2^, and 108 mJ/cm^2^	Contamination rate of patient rooms; Optimal UVC irradiation dose for SARS-CoV-2 environmental disinfection	Date: Not provided **Location:** Taiwan **Sample collection:** Samples were collected on the day after admission and 48 h – 72 h. The patient performed the usual daily activities without any environmental cleaning. The most frequently touched surfaces in their single independent ward of the hospital were selected with 12 points. Environmental samples were collected before irradiation and after 5 s, 10 s, 15 s and 20 s, for SARS-CoV-2 using sterile nasal swabs and analysed using RT-PCR testing. A sample from each equipment surface was collected at three different positions to increase the positive sampling rate. Specific RT-PCR targeting of RNA-dependent RNA polymerase (RdRP), E, and N genes was used to detect the presence of SARS-CoV-2. Room characteristics: Not provided

UVC, ultraviolet C; SARS-CoV-2, severe acute respiratory syndrome coronavirus-2; COVID-19, coronavirus disease 2019; CtV, cycle threshold values; ICU, intensive care unit; RNA, ribonucleic acid; RT-PCR, real-time reverse transcriptase-polymerase chain reaction; US, United States; LTCF, long-term care facility; RdRP, RNA-dependent RNA polymerase.

Among the included studies, one was a non-randomised study with a comparison arm, the other two were before and after non-control studies.^21,22,23^ Details of the included and excluded studies are presented in Online Appendix 1 Table 1. Two of the studies were carried out in hospitals; one in the United States and the other in Taiwan.^21,22^ One was carried out in an isolation room for COVID-19 patients in Taiwan.^23^

One of the studies sampled rooms where COVID-19 patients with a nasal swab SARS-CoV-2 cycle threshold values (CtV) < 25 were admitted for at least 3 days and collected samples using sterile throat swabs from three different locations on the same surface.^22^ Samples were collected every week. In another study, patients who tested positive for SARS-CoV-2 infection with an average cycle threshold values (CtV) of 19 were used to create a contamination environment, similar to a ward in the hospital.^23^ Environmental samples were collected from frequently touched surfaces 48 h – 72 h later, before and after 5 s, 10 s, 15 s and 20 s of UV-C irradiation. The other study collected samples from stationary near-patient, high touch surfaces in an acute care hospital (ACH) using swabs. The samples were collected immediately after SARS-CoV-2 infected patients were discharged or transferred from of their room and the hospital’s cleaning procedure was carried out. Surfaces included bed rails, call button or remote control, in-room telephone, over bed tray table, sink and soap dispenser, chair, windowsill and the floor.^21^

### Intervention

There was no study that compared UVGI with standard cleaning methods such as wiping and spraying. The included studies compared UV-C applications at various wavelengths and exposure times. In one study, the effect of UVC was tested before and after disinfection of the surfaces by wiping with a non-bleach sporicidal disinfection containing hydrogen peroxide and peracetic acid (OxyCide) before exposure to UV-C irradiation. The intervention involved the exposure of UV-C irradiation at 60 000 mJ/cm^2^ compared to either electrostatic spraying (Clorox Total 360) only or UV-C irradiation plus electrostatic spraying. The study did not provide the wavelength of irradiation.^21^ In another study, irradiation was performed at 254 nm by a hyperlight disinfection robot with an energy dose of 42 mJ/cm^2^, 84 mJ/cm^2^, 126 mJ/cm^2^, 168 mJ/cm^2^ and 252 mJ/cm^2^ for 5 min, 10 min, 15 min, 20 min and 30 min of exposure and an estimated width of 3 m.^22^ A Care222 U+ handheld lamp emitting UVC irradiation at 222 nm for 5 s, 10 s, 15 s and 20 s with an energy dose of 27 mJ/cm^2^, 54 mJ/cm^2^, 81 mJ/cm^2^ 108 mJ/cm^2^ with a fixed length of 2 cm was used in the third study.^23^

The risk of bias was judged as low for all the domains of all the studies except for the biases because of the measurement of outcome and selection of the reported result (Online Appendix 1 Table 3). These were judged as moderate. It is probable that investigators who analysed the samples were aware of the source of the samples as there no information was provided on efforts to ensure that staff were blinded to the source of the samples. The studies provided no information on the study protocol and so may not be free from bias due to selective reporting of outcomes.

### Effects of intervention

None of the included studies assessed the effect on the risk of hospital-acquired SARS-CoV-2 infection. All three studies assessed the effectiveness of disinfection on surface decontamination via the presence/absence of viral RNA using real-time polymerase chain reaction (RT-PCR) pre and post UV-C irradiation.

#### Clearance of severe acute respiratory syndrome coronavirus-2 virus on surfaces in healthcare facility

**FIGURE 2:**
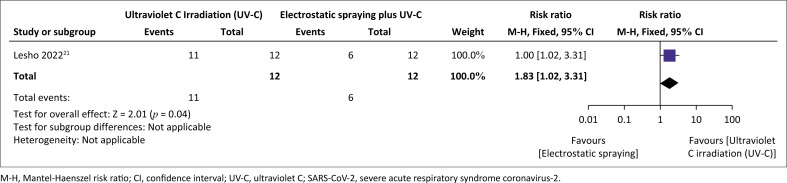
Effect of ultraviolet C irradiation compared to electrostatic spraying on the clearance of severe acute respiratory syndrome coronavirus-2 virus on surfaces in healthcare facility.

**FIGURE 3:**
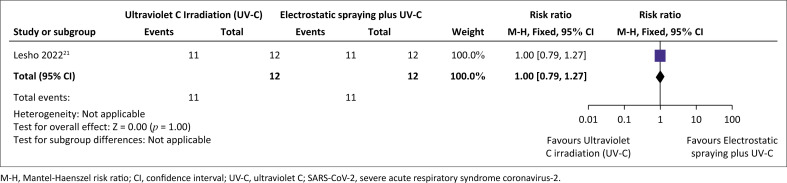
Effect of ultraviolet C irradiation compared to electrostatic spraying plus ultraviolet-irradiation on the clearance of severe acute respiratory syndrome coronavirus-2 virus on surfaces in healthcare facility.

Before pre-cleaning with a non-bleach sporicidal disinfectant, there was a 50% reduction in the number of RNA-positive surfaces following UVC irradiation, compared to electrostatic spraying. Post-cleaning, a 90% reduction of RNA-positive surfaces was observed between UV-C irradiation and electrostatic spraying relative risk (relative risk [RR]: 1.83, 95% confidence interval [Cl]: 1.02–3.31).^21^ This resulted in an 83% reduction in the proportion of RNA-positive surfaces (from 50% RNA-positive pre-cleaning to 8.3% post-cleaning) when UV-C irradiation was used (Figure 2).^21^ No difference was observed when UV-C irradiation only was compared with electrostatic bleach application plus UV-C irradiation pre- and post-cleaning (Figure 3).

The other two studies measured the dose-related effect of varying wavelength of UV-C irradiation. One study reported a contamination rate of 41.7% (*n* = 5/12) before irradiation. Following UV-C irradiation (254 nm), there was a decrease in the number of locations with residual viral RNA; 3 out of 12 (25%), 1 out of 12 (8.3%) and 0 out of 12 (0%) at 5 min, 10 min and 15 min of irradiation, respectively (*p* = 0.005).^22^ The contamination rates immediately after the patients were discharged in the different units were 15.7%, 2.6% and 0% in the different units sampled in the hospital; the ward, ICU and buffer area respectively.^22^ The other study reported a contamination rate of 33.3% (*n* = 16/48) following patient discharge.^23^ Four locations were sampled in the three isolation wards (*n* = 12). Before irradiation, 25% (*n* = 3/12) were positive for residual viral RNA. Following 222 nm UVC irradiations, there was no change at 5 s post-exposure (*n* = 3/12; 25%), but contamination rate decreased to 8.3% (*n* = 1/12) and 0% (*n* = 0/12) after 10 s and 15 seconds, respectively.^23^ The mean CtV for viral RNA in both studies ranges between 30 and 35.^22,23^

#### Optimal irradiation dose/time

Two studies assessed the above outcome.^22,23^ One study reported complete clearance of viral RNA at a wavelength of 254 nm (UVGI) after 15 min of exposure with an estimated energy dose of 125 mJ/cm^2^.^22^ The other study reported complete clearance of viral RNA at far UV-C (222 nm), after 15 seconds of exposure with an estimated energy dose of 81 mJ/cm^2^.^23^

#### Adverse effects associated with the use of ultraviolet germicidal irradiation in healthcare setting

None of the studies assessed adverse effects associated with the use of UVGI.

#### Quality of the evidence

Overall, the certainty of the evidence that UVGI may result in the reduction of a SARS-CoV-2 virus contaminated surface in a health facility was rated as very low across all studies. The studies were downgraded for one or more of the following reasons: insufficient information to assess study design, indirectness because of the use of a non-standard cleaning comparator, small sample side and/or confidence intervals that included the null value. The summary of evidence tables are available in the Online Appendix 1 Table 4.

## Discussion

### Summary of main results

The systematic review sought to determine the effectiveness of UVGI as an environmental (surface) cleaning measure in healthcare settings regarding the prevention of COVID-19 virus infection. An exhaustive literature search yielded three studies, two of which were before and after study, and one was a non-randomised controlled study. None of the studies assessed UVGI and its effect on hospital-acquired SARS-CoV-2 infection. The review found that the use of UVGI as an environmental cleaning measure for surfaces in healthcare settings where patients with COVID-19 are managed reduced viral contamination, but to varying degrees. There was wide variation in the mode of delivery of the intervention. One study comparing the proportion of detectable viral RNA on surfaces indicated a lower proportion of residual surface contamination from UVGI compared to electrostatic spraying but found no difference when UVGI was compared to both UVGI and electrostatic spraying.^21^ Two of the included studies assessed the dose-related effect of UVGI and reported no detectable viral RNA on surfaces following 15 s and 15 min of UV-C irradiation at wavelengths 222 nm and 254 nm, respectively.^22,23^ The certainty of the evidence was rated as very low across all studies.

### Overall completeness and applicability of evidence

Although existing literature suggests the potential benefits of UVGI in reducing the risk of SARS-CoV-2 infection from contaminated surfaces, the review did not find studies that directly evaluate its impact on humans. Specifically, there is a lack of evidence regarding the effect of UVGI in reducing the morbidity rate of SARS-CoV-2 infection among non-COVID-19 patients and/or staff. The studies included in the review provide indirect evidence of potential risk by sampling surfaces in areas where patients were treated or isolated, demonstrating that UVGI irradiation can effectively clear residual viral particles. Only one study measured the effect within the optimal UVGI range at a wavelength of 254 nm. The applicability of the findings of this review should be approached with caution given the substantial heterogeneity across different studies in terms of how the intervention was implemented and the variations in the structure and size of the sampled rooms. Additionally, it is crucial to note that the detection of viral RNA does not equate to infectiousness or the presence of an active disease as polymerase chain reaction (PCR) can identify remnants of the virus even after it has been inactivated. Most cycle threshold (Ct) values for the studies were greater than 31, suggesting the possible presence of a non-transmissible virus.

Ultraviolet germicidal irradiation is a well-established method for disinfection and environmental control of various infections, including tuberculosis.^7,9^ However, there are health risks associated with UV-C irradiation. Prolonged or direct exposure may lead to conditions such as melanoma, erythema and eye disorders.^24,25^As such, UVGI systems are primarily used in settings where persons can be evacuated during use, such as patient rooms after discharge and in operating rooms, where they complement routine disinfection practices. To mitigate the associated health risks, one of the studies included explored UVC at a wavelength of 222 nm, which has been shown to be safe in humans at a dose of 500 mJ/cm^2^ while still maintaining its bactericidal effect.^24^ The study demonstrated results comparable to studies utilising UV-C irradiation at a higher wavelength such as 254 nm.^23^

While UVGI is effective at reducing surface contamination in smaller, low-traffic spaces like patient rooms, its effectiveness may be diminished in larger or high-traffic areas. In high-traffic areas including hospital corridors or waiting rooms, UVGI may not be the most ideal method for surface disinfection. These areas are constantly exposed to new contaminants, dirt and dust. Dirt and dust can create a layer of organic matter, blocking pathogens and reducing the disinfecting power of UVGI.^26^ To enhance UVGI efficacy, surfaces must be relatively clean, which is why studies have reported greater reduction in RNA contamination on surfaces after cleaning with disinfectant.^21,27^

Additionally, rooms with a large equipment or irregular surfaces on furniture or equipment can prevent UVC light from reaching all areas. Larger rooms or open spaces may require multiple UVGI units. The room’s ventilation rate also plays a role in UVGI efficiency high ventilation rates or poorly mixed airflows can reduce the exposure of particles to UVC radiation, decreasing its effectiveness.^28,29^

The humidity levels in the room and the type of surfaces being disinfected have been shown to affect the effectiveness of UV-C irradiation. High humidity reduces the intensity and penetrating power of UV-C irradiation.^28^ Surfaces with silicon, such as washbasins or wet areas in healthcare setting with high humidity, may require longer exposure times for effective disinfection.^22^ Additionally, UV-C light bulbs should be checked regularly, to ensure they are free from dusts to ensure optimal UV-C intensity for germicidal action. As the bulbs age, their intensity decreases, which can reduce their effectiveness.^25^

### Strengths and limitations

The review includes an exhaustive search of several databases without study type or language restrictions. In addition, the review followed the robust and rigorous systematic review methodology developed and recommended by Cochrane.^19^ A limitation of this review is the absence of comparative studies. The restricted search period may have resulted in the exclusion of earlier publications, and the lack of an extensive search for grey literature may have limited the number of included studies. We attempted to minimise bias during the review process by performing study screening, data extraction and risk of bias assessment by two independent reviewers. Other evidence available on transmission in healthcare settings from contaminated surfaces were not captured was because of the specific focus of the review on SARS-CoV-2 infection. Furthermore, the studies incorporated into the analysis had a small sample size when considering the number of surfaces that were sampled.

## Conclusion

The review provides indirect evidence on the use of UVGI in reducing the spread of the SARS-CoV-2 virus, with UV-C irradiation demonstrating effectiveness in reducing viral presence on surfaces in healthcare setting. The required irradiation exposure time varied depending on the UV-C wavelength, but overall, UVGI shows promise in minimising surface contamination. However, combining UVGI with other cleaning methods may be more effective in reducing the spread of the virus in healthcare settings. While UVGI has potential benefits, further research is necessary to determine its impact on reducing the risk of SARS-CoV-2 infections among healthcare personnel.

Several studies have examined the use of UVGI for SARS-CoV-2 inactivation on contaminated surfaces and these findings are primarily based on laboratory settings.^29^ While laboratory results provide useful insights into surface contamination, there is limited evidence regarding UVGI’s role in reducing hospital-acquired SARS-CoV-2 infections. Transmission risk is influenced by numerous factors, including the healthcare setting, cleaning practices and adherence to protocols by both healthcare workers and patients. The studies were conducted over short periods with variation in UV wavelength and exposure times, therefore, more research is needed to establish definitive evidence on its effectiveness and best practices for its use in real-world healthcare environments.

## Appendix 1

**TABLE 1-A1:** Search strategy and output.

1.	Ultraviolet Rays/ or Ultraviolet Therapy/
2.	(ultraviolet or ultra violet or UV or UVGI or UVC).ti,ab,kf.
3.	(actinic adj3 (ray* or radiat* or irradiat*)).ti,ab,kf.
4.	(germicid* adj3 lamp*).ti,ab,kf.
5.	or/1-4
6.	limit 5 to covid-19
7.	exp Animals/ not Humans/
8.	6 not 7
9.	(comment or editorial or newspaper article).pt.
10.	8 not 9
11.	exp ultraviolet radiation/
12.	ultraviolet germicidal irradiation/
13.	ultraviolet irradiation/
14.	ultraviolet phototherapy/
15.	(ultraviolet or ultra violet or UV or UVGI or UVC).ti,ab,kw.
16.	(actinic adj3 (ray* or radiat* or irradiat*)).ti,ab,kw.
17.	(germicid* adj3 lamp*).ti,ab,kw.
18.	or/11-17
19.	limit 18 to COVID-19
20.	exp animal/ not exp human/
21.	19 not 20
22.	editorial.pt.
23.	21 not 22
24.	(ultraviolet or ultra violet or UV or UVGI or UVC).mp.
25.	(actinic adj3 (ray* or radiat* or irradiat*)).mp.
26.	(germicid* adj3 lamp*).mp.
27.	or/24-26
28.	(nCoV* or 2019nCoV or 19nCoV or COVID19* or COVID or SARS-COV-2 or SARSCOV-2 or SARS-COV2 or SARSCOV2 or SARS coronavirus 2 or Severe Acute Respiratory Syndrome Coronavirus 2 or Severe Acute Respiratory Syndrome Corona Virus 2).mp.
29.	(longCOVID* or postCOVID* or postcoronavirus* or postSARS*).mp.
30.	((Wuhan or Hubei) adj5 pneumonia).mp.
31.	(coronavirus* or corona virus* or betacoronavirus* or CoV or HCoV).mp.
32.	28 or 29 or 30 or 31
33.	limit 32 to yr= ‘2019 –Current’ *[COVID-19 filter adapted from CADTH Search Strings]*
34.	27 and 33
35.	10 use medal
36.	23 use emczd
37.	34 use cctr
38.	34 use coch
39.	35 or 36 or 37 or 38
40.	remove duplicates from 39

Note: Embase Classic+Embase 1947 to 31 August 2022, Ovid MEDLINE(R) ALL 1946 to 31 August 2022, EBM Reviews – Cochrane Central Register of Controlled Trials July 2022, EBM Reviews – Cochrane Database of Systematic Reviews 2005 to 31 August 2022.
